# The First Report of Differences in Gut Microbiota Composition between Obese and Normal Weight Iranian Subjects

**DOI:** 10.29252/ibj.24.3.148

**Published:** 2019-12-14

**Authors:** Fateme Ettehad Marvasti, Arfa Moshiri, Mina Sadat Taghavi, Soheil Riazi, Majid Taati, Seyedeh Fatemeh Sadati, Azadeh Ghaheri, Morteza Masoomi, Farzam Vaziri, Abolfazl Fateh, Pejman Rohani, Samira Tarashi, Andrea Masotti, Sara Ahmadi Badi, Seyed Davar Siadat

**Affiliations:** 1 Microbiology Research Centre, Pasteur Institute of Iran, Tehran, Iran;; 2 The Islamic Azad University, Science and Research Branch, Tehran, Iran;; 3 Gastroenterology and Liver Diseases Research Center, Research Institute for Gastroenterology and Liver Diseases, Shahid Beheshti University of Medical Sciences, Tehran, Iran;; 4 Laboratory of Experimental Therapies in Oncology, IRCCS Istituto Giannina Gaslini, Genoa, Italy;; 5 Departments of Epidemiology and Reproductive Health, Reproductive Epidemiology Research Center, Royan Institute for Reproductive Biomedicine, ACECR, Tehran, Iran;; 6 Mycobacteriology and Pulmonary Research Department, Pasteur Institute of Iran, Tehran, Iran;; 7 Department of Pediatric Gastroentrology and Hepatology, Mofid Children's Hospital, Shahid Beheshti University of Medical Sciences, Tehran, Iran;; 8 Research Laboratories, Bambino Gesù Children's Hospital-IRCCS, Rome, Italy;; 9 Endocrinology and Metabolism Research Center, Endocrinology and Metabolism Clinical Sciences Institute, Tehran University of Medical Sciences, Tehran, Iran

## Abstract

**Background:**

Obesity is a complex disorder influenced by various genetic and environmental factors. It has been shown that gut microbiota, which colonizes gastrointestinal tract, has a substantial role as an environmental factor in the pathophysiology of obesity. Since the composition of gut microbiota alters with regard to different criteria, such as ethnicity, geographical location, diet, lifestyle, age, and gender, we aimed to determine F/B ratio and the abundance of important gut microbiota members, *A. muciniphila*, *F. prausnitzii, Roseburia, Bifidobacterium*, and* Prevotella* in Iranian obese and normal weight individuals, for the first time.

**Methods:**

In this study, 50 normal and 50 obese subjects were recruited and classified based on their BMI into normal weight and obese groups. Stool samples were collected. Following DNA extraction from the samples, qPCR was conducted based on 16s rDNA universal primers. Finally, the correlation between the bacterial abundance and obesity was analyzed by statistical analyses.

**Results:**

We observed a significant increase of F/B ratio in the obese group, compared to the normal weight group (*p *= 0.002). Although *A. muciniphila *(*p* = 0.039) and* Bifidobacterium *(*p *= 0.049) abundance significantly decreased, the abundance of *F. prausnitzii* (*p *= 0.046) significantly elevated with BMI increase in the studied groups.

**Conclusion:**

Owing to the importance of the gut microbiota composition in obesity development, determination and targeted restoration of gut microbiota pattern could be valuable in the control and treatment of obesity in certain populations.

## INTRODUCTION

Obesity is a global health problem due to change in people’s life style. Various factors, including genetic and environmental factors, are involved in the pathophysiology of obesity^[^^1^^-^^3^^]^. Gut microbiota has been known as an important environmental factor for inducing and developing obesity. After birth, the gastrointestinal tract is colonized by a complex and dynamic microbial community, which is called gut microbiota. The composition of gut microbiota depends on multiple factors, including genetic background, mode of delivery, nutrition, antibiotic consumption, physical activity, geographical distribution, ethnicity, age, gender, lifestyle, and others^[^^4^^-^^6^^]^. This microbial community settles down during 2-3 years of life and consists of bacteria, archaea, protozoa, fungi, and viruses. Bacteria are dominant in this microbial population where the *Firmicutes* and *Bacteroidets* phyla make up the most frequency of gut microbiota. Also, *Actinobacteria*, *Proteobacteria*, and *Verrucomicrobia* are constituents of gut microbiota with low frequency^[^^7^^,^^8^^]^. The gut microbiota and its metabolites have determinative role in health and diseases due to the fact that they have significant potential in host, including regulation of inflammatory responses, energy homeostasis, and glucose/lipid metabolism. Therefore, any change in gut microbiota composition, which is termed dysbiosis, can lead to disruption in host functions and development of metabolic disorders, including obesity and type 2 diabetes . In this regard, determination of altered gut microbiota composition is an inevitable part of etiological recognition of obesity^[^^2^^,^^9^^,^^10^^]^.

Obesity is associated with low-grade inflammation, insulin resistance, increased weight gain, and fat deposition^[^^2^^,^^11^^]^. It has been documented that high-fat diet induces dysbiosis, which favors the increase of energy harvest from diet, deregulation of immune responses, and metabolic pathways^[^^1^^,^^2^^,^^12^^]^. Hence, to achieve healthy state, the delicate arrangement of gut microbiota composition in the gastrointestinal tract is required. In this regard, many studies have shown that F/B ratio increases in obese subjects, and changes in F/B ratio have a significant role in calorie intake and have a direct correlation with obesity^[^^13^^,^^14^^]^. 

Currently, numerous investigations have shown that anaerobic intestinal commensal bacteria such as *A. muciniphila,* *F. prausnitzii*, *Roseburia*,* Bifido-bacterium*, and* Prevotella* have significant role in the gut microbiota-host interactions, including influence on host metabolism and immune system through anti-inflammatory properties. Thus, their relative abundance could be a potential health biomarker^[^^15^^-^^19^^]^. 

As mentioned above, dysbiosis is critical starting point in developing obesity and related complications (type 2 diabetes, cardiovascular disease, nonalcoholic fatty liver disease, etc.). Also, F/B ratio*, A.* *muciniphila, F. prausnitzii, Roseburia, Bifidobacterium*, and *Prevotella* influence the pathophysiology of metabolic disorders. As the gut microbiota is under the influence of different factors such as ethnicity, diet, lifestyle, and geographical distribution, we decided to investigate the relative abundance of these bacteria in obese Iranian population, which would be the first report in this context, to the best of our knowledge. For this purpose, fecal samples from Iranian subjects were collected and analyzed using qPCR based on 16s rDNA gene of targeted bacteria. We aimed to determine the correlation between the abundance of the aforementioned bacteria and BMI among our studied population.

## MATERIALS AND METHODS

**Study population**

A total of 100 adult Iranian individuals (aged between 20 and 60 years) were selected for this study during October 2016 to December 2017. The subjects were equally grouped into normal weight group with BMI between 18.5 and 24.9 kg/m^2^ and obese group with BMI above 25 kg/m^2^. Exclusion criteria included the use of corticosteroids, antibiotics, alcohol, smoking, significant infection, and gastrointestinal diseases. 

**Fecal sampling and DNA extraction**

Subjects were asked to collect their stool samples in a conventional laboratory plastic container dedicated for fecal sampling. The samples were immediately transferred to the laboratory in cold chain storage. These samples were stored at -80 °C (fresh frozen) upon arrival until further processing. DNA was extracted from the samples using QIAamp DNA stool mini kit (Qiagen, Hilden, Germany) according to manufacturer’s instructions. The quality and quantity of the extracted DNA was analyzed by agarose gel electrophoresis and NanoDrop ND-8000 (Thermo Scientific, USA), respectively^[^^20^^]^.

**qPCR analyses**

The abundance of bacteria was analyzed using qPC based on SYBER green method (LightCycler® 96 SW 1.1; Roche, Germany)^[13,20,21]^. Each 20 µl of qPCR reaction was composed of SYBR Premix Ex Taq II 

**Table 1:** 16S rRNA gene specific primers for the studied bacterial group/species

** Target organism**	**Forward (5´ to 3´)**	**Reverse (5´ to 3´)**	**Ref.**
Firmicutes	TGAAACTYAAGGAATTGACG	ACCATGCACCTGTC	^[^^13^^]^
Bacteroidetes	AAACTCAAAKGAATTGACGG	GGTAAGGTTCCTCGCGCTAT	^[^^13^^]^
*muciniphila*	CAGCACGTGAAGGTGGGGAC	CCTTGCGGTTGGCTTCAGAT	^[^^20^^]^
*F. prausnitzii*	GGAGGAAGAAGGTCTTCGG	AATTCCGCCTACCTCTGCACT	^[^^21^^]^
Prevotella	CACCAAGGCGACGATCA	GGATAACGCCYGGACCT	^[^^47^^]^
Roseburia	TACTGCATTGGAAACTGTCG	CGGCACCGAAGAGCAAT	^[^^47^^]^
Bifidobacterium	CTCCTGGAAACGGGTGG	GGTGTTCTTCCCGATATCTACA	^[^^48^^]^
*Escherichia coli*	CATTGACGTTACCCGCAGAAGAAGC	CTCTACGAGACTCAAGCTTGC	^[^^49^^]^

(RR820L; Takara, Japan), 0.5 µl of each of the specific 16s rDNA primers (Table 1), and 1 µ1 of the DNA template. Each qPCR reaction was performed in duplicate using LightCycler^®^ 8-Tube Strips (white; Roche). The amplification program was designed

according to the appropriate annealing temperature: 1 cycle of 95 °C for 60 s, followed by 40 cycles of denaturation at 95 °C for 5 s, annealing at 55 °C for 30 s, and extension at 72 °C for 30 s. Melting curve analysis was carried out after amplification to control the specificity of PCR reaction, followed by 1 cycle at 95 °C for 5 s, 60 °C for 60 s, and 95 °C for 1 s. 

**Standard curve**

The abundance of bacteria was calculated as previously described^[^^22^^]^. Briefly, the standard curve was prepared using serial dilutions of DNA from standard strain *Escherichia coli*. This curve allows us to calculate DNA concentration of each bacterium from fecal samples. The standard curve is graphically represented as a semi-log regression line plot of CT value vs. log of DNA concentration.

**Statistical analyses**

In this study, categorical variables are presented as numbers (percent) and continuous variables as mean ± SD. Independent *t*-test was employed to assess mean differences between the normal and obese groups. Chi-square analysis was used for qualitative data, and K-S analysis was applied to control the normal distribution of the data. Linear regression model and Pearson’s correlation coefficient were performed to determine correlation between BMI and the abundance of two bacteria phyla, *Bacteroidetes* and *Firmicutes*. Statistical analyses were conducted using SPSS version 24.0 (SPSS Inc., Chicago, IL, USA). All statistical tests were 2-tailed, and a *p* < 0.05 was considered statistically significant. 

**Ethical statement**

The above-mentioned sampling protocols were approved by the National Institute for Medical Research Development (NIMAD, Tehran, Iran; ethical code: IR.NIMAD.REC.1395.043). Written informed consents were provided by all the patients. 

## RESULTS

**Demographic characteristics of the study population**

Adult subjects were divided, based on BMI, into two groups: normal weight (50%) and obese (50%). Characteristics of the subjects are shown in Table 2. Obese group consisted of 32 overweight subjects with BMI between 25 and 29.9 kg/m^2^ and 18 subjects with BMI above 30 kg/m^2^. There were not significant differences in age, gender, and height between the two studied groups. 

**Table 2:** Characteristics of obese and normal weight adults under study

**Characteristics**	**Obese**	**Normal weight**
Subjects	50	50
Gender (male/female)	25/25	25/25
Age (y)	38.76 ± 1.76	38.74 ± 1.41
Weight (kg)	83.92 ± 1.89	64.8 ± 1.24
Height (m)	1.69 ± 0.015	1.69 ± 0.012
BMI (kg/m2)	29.36 ± 0.50	22.4 ± 0.26
BMI s.d. score	3.55	1.86

**Table 3:** Mean abundance of each phylum across each of the BMI categories

**Phylum**	**BMI ** **index**		***p value***
	**18.5-24.9**	**≥25**	
*Firmicutes*	6.59 ± 0.19	7.15 ± 0.18	0.045
*Bacteroidetes*	4.92 ± 08.0	4.64 ± 10.0	0.040
F/B	1.66 ± 0.18	2.5 ± 0.19	0.002

**F/B ratio**

**Fig. 1:**
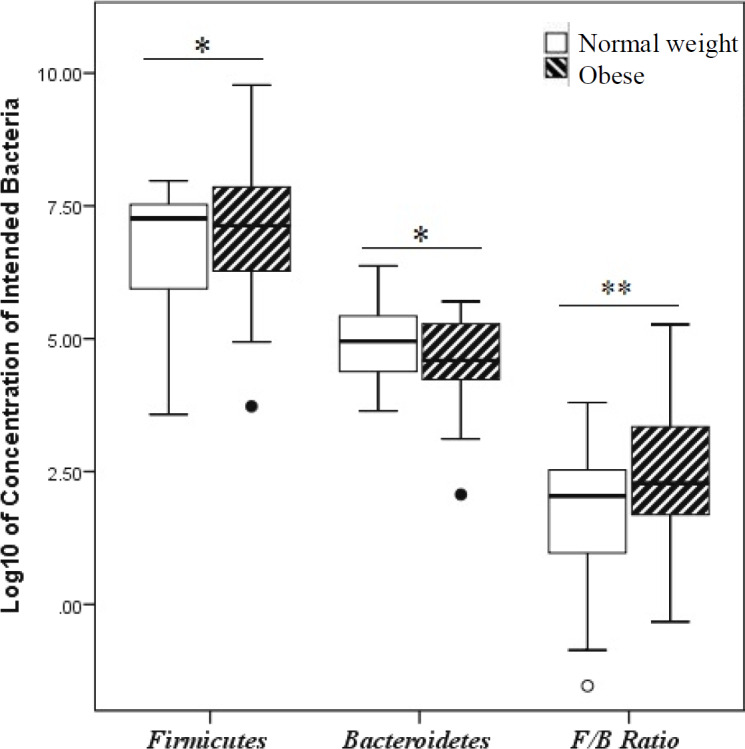
F/B ratio of obese and normal weight Iranian subjects detected by qPCR. ^*^p < 0.05; ^**^p < 0.01

In Table 3, the mean abundance of *Firmicutes*, *Bacteroidetes*, and F/B are presented. The results demonstrated that *Firmicutes* and *Bacteroidetes* abundance significantly increased and decreased in the obese group and the control, respectively (Fig. 1). Besides, the F/B ratio was significantly higher in the obese (*p *= 0.002) than control group.

**Quantification and comparison of important gut microbiota memebers**

**Fig. 2:**
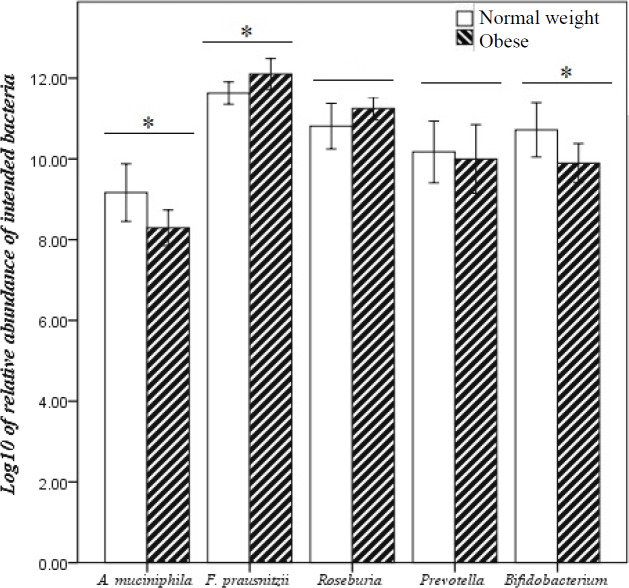
Comparison of the abundance of important gut microbiota members in normal weight and obese Iranian subjects detected by qPCR. Data of qPCR are expressed as mean log_10_ CFU/g. Error bars 95% CI. ^*^p < 0.05

The concentrations of *A. muciniphila*, *F. prausnitzii, Roseburia, Prevotella*, and *Bifidobacterium *were quantified in fecal samples of the studied groups. The highest and lowest concentrations of targeted bacteria were 8.69 × 10^13^ and 1.88 × 10^5 ^CFU/g for *A. muciniphila*, 9.71 × 10^13 ^and 1.83 × 10^7 ^CFU/g for *F. prausnitzii, *2.06 × 10^13 ^and 4.95 × 10^1^ CFU/g for *Roseburia*, 7.84 × 10^14 ^and 8.69 × 10^2 ^CFU/g for *Prevotella*, and 4.52 × 10^15 ^and 1.19 × 10^1 ^CFU/g for *Bifidobacterium* in normal weight and obese groups, respectively (Table 4). In order to find a correlation between the bacterial abundance and BMI, differences in gut microbiota composition between the two groups were analyzed. Our results demonstrated that *A. muciniphila* relative abundance significantly decreased in parallel with BMI increase in obese vs. normal weight (*p* = 0.039) groups. Also, a significant reduction of *Bifidobacterium* relative abundance (*p* = 0.049) was observed in the obese group. In contrast, there was a significant increase of *F. prausnitzii* relative abundance in the obese compared to the normal weight (*p *= 0.460) subjects. Although the relative abundance of *Roseburai* (*p* = 0.170) and *Prevotell* (*p *= 0.756) increased and decreased with BMI increase, respectively, no significant correlation was found between their frequency and the studied groups (Fig. 2). 

## DISCUSSION

The dominant roles of gut microbiota in pathophysiology of obesity has been currently evidenced^[^^23^^]^. Several studies have shown that the imbalance of energy homeostasis, low-grade inflammation, and insulin resistance are important determinants, which result from dysbiosis and lead to the negative regulation of host metabolism^[^^24^^]^. Due to the fact that gut microbiome exerts crucial functions such as the influence on energy harvest from diet, and anti-inflammatory and metabolism regulation, any change in gut microbiota composition can induce and develop obesity^[^^24^^,^^25^^]^. According to the importance of gut microbiota composition in obesity and its uniqueness in each population, we studied, for the first time, the differences of F/B ratio and the relative abundnce of *A. muciniphila*,* F.* prausnitzii*, Roseburia*,* Bifidobacterium*, and *Prevotella* in normal weight and obese Iranian subjects. 

**Table 4:** Relative abundance of *A. muciniphila*, *F. prausnitzii*,* Roseburia*,* Prevotella*, and* Bifidobacterium*

	***A. muciniphila*** ** (CFU/g)**	***F. prausnitzii*** **(CFU/g)**	***Roseboria*** **(CFU/g)**	***Prevotella*** **(CFU/g)**	***Bifidobacterium*** **(CFU/g)**
Mean	1.93 × 10^12^	4.90 × 10^12^	1.05 × 10^12^	4.07 × 10^13^	6.69 × 10^13^
Std. Error of Mean	1.02 × 10^12^	1.13 × 10^12^	2.44 × 10^11^	1.43 × 10^13^	4.65 × 10^13^
Std. Deviation	1.02 × 10^13^	1.13 × 10^13^	2.44 × 10^12^	1.43 × 10^14^	4.65 × 10^14^
Minimum	1.88 × 10^5^	1.83 × 10^7^	4.95 × 10^1^	8.69 × 10^2^	1.19 × 10^1^
Maximum	8.69 × 10^13^	9.71 × 10^13^	2.06 × 10^13^	7.84 × 10^14^	4.52 × 10^15^

Various animal and human studies have revealed the increase of *Frimicutes* and decrease of *Bacteroidets* concentrations (increase of F/B ratio) in obese vs. normal subjects^[^^26^^-^^29^^]^, which is in line with our study. Dominancy of Firmicutes, which is enriched by bacterial genes related to nutrient transporters and primary fermentation enzymes, could be explained by elevated calories absorption and weight gain during obesity^[^^30^^,-^^32^^]^. Higher frequency of bacterial genes responsible for carbohydrate metabolism, which belongs to *Bacteroidetes*, has been reported in Turnbaugh *et al*.'s research of lean and obese-twin gut microbiome^[^^30^^]^. Nevertheless, there are inconsistent results of F/B ratio. In this regard, Andoh *et al*.^[^^33^^]^ did not observed any difference in the F/B ratio between the obese and non-obese Japanese groups. 

Recently, the significant roles of some intestinal anaerobic commensal bacteria, such as *A. muciniphila*, F. prausnitzii, *Bifidobacterium*, *Roseburia*, and *Prevotella*, have been illustrated in gut microbiota-host interaction^[^^24^^,^^25^^]^. One important feature of these bacteria is the production of short-chain fatty acids, which have various functions, including the regulation of gut barrier integrity, regulation of metabolism, and inflammation. Thus, there is a correlation between the abundance of mentioned bacteria and obesity^[^^24^^,^^25^^,^^34^^-^^36^^]^. 

Increasing body of evidence in animal and human studies has demonstrated an inverse correlation of *A. muciniphila* abundance with obesity^[^^36^^,^^37^^]^. In agreement with other studies, *A. muciniphila *abundance significantly reduced in Iranian obese subjects in comparison with normal weight subjects. 

This reduction of *A. muciniphila *abundunce is associated with impaired metabolic status during obesity, since it has many health promoting potentials, including regulation of glucose metabolism, blood lipid concentration, and fat distribution^[^^15^^]^. 

The genus *Bifidobacterium* has been shown to have beneficial health effects due to the effect on the gut barrier and immune system^[^^38^^]^. The association of *Bifidobacterium* abundance and obesity has been studied in several studies^[^^39^^-^^42^^]^. Ignacio *et al.*^[^^43^^]^ have reported the negative correlation between the abundance of *Bifidobacterium* and BMI and showed increased abundance of *Bifidobacterium* spp. in the lean group. Similarly, we identified significant negative correlation of Bifidobacterium abundance with BMI increase. 

*There are various studies that inconsistently reported that the *F. prausnitzii *abundance is associated with obesity*^[^^44^^-^^46^^]^*. In accordance with the report of gut microbiota composition in obese Indian children*^[^^46^^]^*, the abundance of *F. prausnitzii* significantly increased along with BMI increase in Iranian subjects.* However, Feng *et al*.^[^^45^^]^ did not observe any significant difference of F. prausnitzii *levels between obese and normal Chinese subjects.*

Taken together, our results demonstrated a significant increase of F/B ratio and reduction of *A. muciniphila *and Bifidobacterium in obese Iranian subjects vs. normal weight individuals. additionally, we observed higher F. prausnitzii abundance in obese subjects. Since gut microbiota composition is established based on various factors, including ethnicity, diet, life style, and geographical location, which induce differences between various populations, it is necessary to determine this composition to design proper strategies for obesity treatment in each targeted population. 
